# Anatomy

**Published:** 1836-07

**Authors:** 


### PART THIRD.

### Selections from Foreign Journals.

### ANATOMY.

*On the Arteries of the Corneal Conjunctiva.* By Dr. Römer, of Vienna.

This paper commences with a full and impartial account of the various opinions which have been entertained by different anatomists regarding the covering of the cornea. A very simple experiment, viz. throwing the eye of an animal into boiling water, shows that the cornea is covered externally by a membrane, which, although it be continued into the conjunctiva, differs remarkably from the latter tunic in physical properties, and is as strikingly different from the proper substance of the cornea. The membrane in question appears to be albuminous; for in this experiment it immediately coagulates and becomes opaque, while the palpebral and sclerotic portions of the conjunctiva suffer no such change. Dr. Römer regards the conjunctiva as a continuation of the common integuments, changed into mucous membrane on the inside of the eyelids, and again, on the anterior part of the sclerotica and on the cornea, transformed into serous membrane. He speaks of the Meibomian and caruncular follicles as the sebaceous glands of the conjunctiva; but, to our view, the Meibomian follicles open through the skin, not through the conjunctiva. It is well known that that portion of the conjunctiva which covers the internal surface of the tarsi or cartilages is villous or papillous, while the remainder of the membrane is comparatively smooth. It is the villi of the former which are so much enlarged and indurated in granular conjunctiva. They are considered by Eble* not as secreting organs, but merely as nervous papillæ, like those of the skin or of the tongue; but the general notion is that they are mucous glands, and this appears to be the opinion also of Dr. Römer.

In the normal state, says Dr. Römer, the conjunctiva lining the inner surface of the eyelids is moderately red, while that portion which covers the eyeball is of a pearl colour, and only towards the angles of the eye presents occasionally some insulated red serpentine streaks, which are blood-vessels. After a tolerably successful injection, the conjunctiva of the eyelids presents a high red colour and a peculiar velvety appearance, bearing a striking resemblance to the Schneiderian membrane covering the septum narium, while from the ocular conjunctiva the pearly hue has vanished, and in its stead we find a rose-red colour, reaching in general only as far as the edge of the cornea. In a completely successful injection, the vessels are prolonged to the centre of the cornea, where they evidently penetrate and are lost in its substance.

The vessels which are distributed to that part of the conjunctiva which covers the anterior portion of the sclerotica and the cornea, are derived, as well as those which belong to the palpebral conjunctiva, from the lacrymal, the superior and inferior palpebral, and the oculo-muscular arteries. The small branches of these arteries form in that part of the conjunctiva which covers the anterior portion of the sclerotica, a superficial and a deep-seated network. The superficial network is derived from numerous branches arising from the superior and inferior palpebral arteries, and from the lacrymal. Dividing, as they proceed, into smaller branches, they run in a serpentine course towards the edge of the cornea, where they anastomose together in arches, and connect themselves with the deep-seated network. The deep-seated network is formed by much smaller vessels, partly derived from

* Ueber den Bau und die Krankheiten der Bindehaut des Auges. Wien, 1828.

the oculo-muscular arteries, and partly from the ciliary, before these penetrate through the sclerotica. The ramifications of the two networks form at the edge of the cornea a vascular wreath, which lies just over the circular venous sinus of the iris. From every part of this wreath arise numerous minute ramifications, running towards the centre of the cornea, each dividing, as they run, into two or three still smaller vessels, which plainly dip into the substance of the cornea.

[We regard Dr. Römer's paper as a valuable contribution to the anatomy of the eye.] *Zeitschrift für die Ophthalmologie*, B. v. H. 1. 1835.

